# Acute Cerebellitis Due to Administration of Durvalumab for Recurrent Endometrial Cancer: A Case Report

**DOI:** 10.7759/cureus.97372

**Published:** 2025-11-20

**Authors:** Hirofumi Kawahara, Kyoko Taketoshi, Taiga Nagano, Takashi Matsuo, Hiroaki Kobayashi

**Affiliations:** 1 Obstetrics and Gynecology, Saiseikai Sendai Hospital, Kagoshima, JPN; 2 Obstetrics and Gynecology, Kagoshima University Hospital, Kagoshima, JPN

**Keywords:** carboplatin-paclitaxel, immune-related adverse events, immunoglobulin, immunotherapy, nystagmus

## Abstract

Immunotherapy with immune checkpoint inhibitors (ICI) has recently been introduced to advanced and recurrent endometrial cancers. The addition of durvalumab to standard carboplatin-paclitaxel (TC) therapy improved outcomes for patients with endometrial cancer. However, immune-related adverse events (irAEs) are being increasingly recognized, and neurological irAEs are rare, particularly those confined to the cerebellum. We present the case of a 55-year-old woman with recurrent endometrial cancer who developed acute neurological symptoms during treatment with TC plus durvalumab therapy. The patient presented with an unsteady gait, and initial brain magnetic resonance imaging excluded cerebral infarction. As her symptoms persisted, the patient was transferred to a tertiary neurology hospital. Neurological examinations revealed truncal ataxia, dizziness, dysarthria, and nystagmus. Based on neurological findings, cerebrospinal fluid and blood analyses, and the clinical course, the patient was diagnosed with immune-related cerebellitis. Chemotherapy was discontinued as the event was graded as a severe (grade 3) irAE. High-dose corticosteroid pulse therapy was administered twice, followed by intravenous immunoglobulin (IVIG), owing to an incomplete response. Despite treatment, nystagmus persisted. Although no disease progression was observed, her quality of life (QOL) was profoundly impaired, and supportive care was initiated.

Neurological irAEs can cause irreversible sequelae and significantly impair QOL. When neurological symptoms are observed during ICI therapy, prompt collaboration with a neurologist and early intervention are essential to minimize long-term disability. This case highlights the importance of vigilance for rare neurological irAEs, such as immune-related cerebellitis in gynecological oncology practice.

## Introduction

The phase Ⅲ DUO-E trial demonstrated that the addition of the immune checkpoint inhibitor (ICI) durvalumab to standard carboplatin-paclitaxel (TC) therapy improved outcomes for patients with endometrial cancer [1]. Durvalumab exerts its effects by blocking programmed death ligand-1 (PD-L1) expression in tumor cells, thereby preventing its interaction with programmed death-1 (PD-1) receptors on T lymphocytes. This inhibition restores antitumor immune surveillance by overcoming immune escape mechanisms [2]. Although this strategy provides substantial survival benefits, a wide spectrum of immune-related adverse events (irAEs) has been reported. Neurological irAEs are particularly rare, with grade ≥3 events observed in fewer than 1% of ICI-treated patients [3]. Neurological irAEs encompass both the peripheral nervous system and the central nervous system (CNS) involvement. Peripheral nervous system manifestations include myositis, myasthenia gravis, and Guillain-Barré syndrome. In contrast, CNS involvement may present as encephalitis, meningoencephalitis, limbic encephalitis, cerebellar encephalitis, aseptic meningitis, or longitudinal extensive transverse myelitis. Cerebellitis represents a particularly rare subtype and poses diagnostic challenges because its symptoms can initially mimic stroke or paraneoplastic neurological syndromes (PNS). In the gynecologic oncology field, the introduction of ICIs has occurred later compared with other oncology fields, resulting in fewer cases of neurological irAEs and limited clinical familiarity with their presentation and management. Herein, we report a case of immune-related cerebellitis in a patient with recurrent endometrial cancer treated with durvalumab and TC chemotherapy. The neurological toxicity was severe, making it challenging to continue chemotherapy. This case illustrates the diagnostic and therapeutic challenges of immune-related cerebellitis and highlights the importance of prompt recognition and multidisciplinary management.

## Case presentation

**Figure 1:**
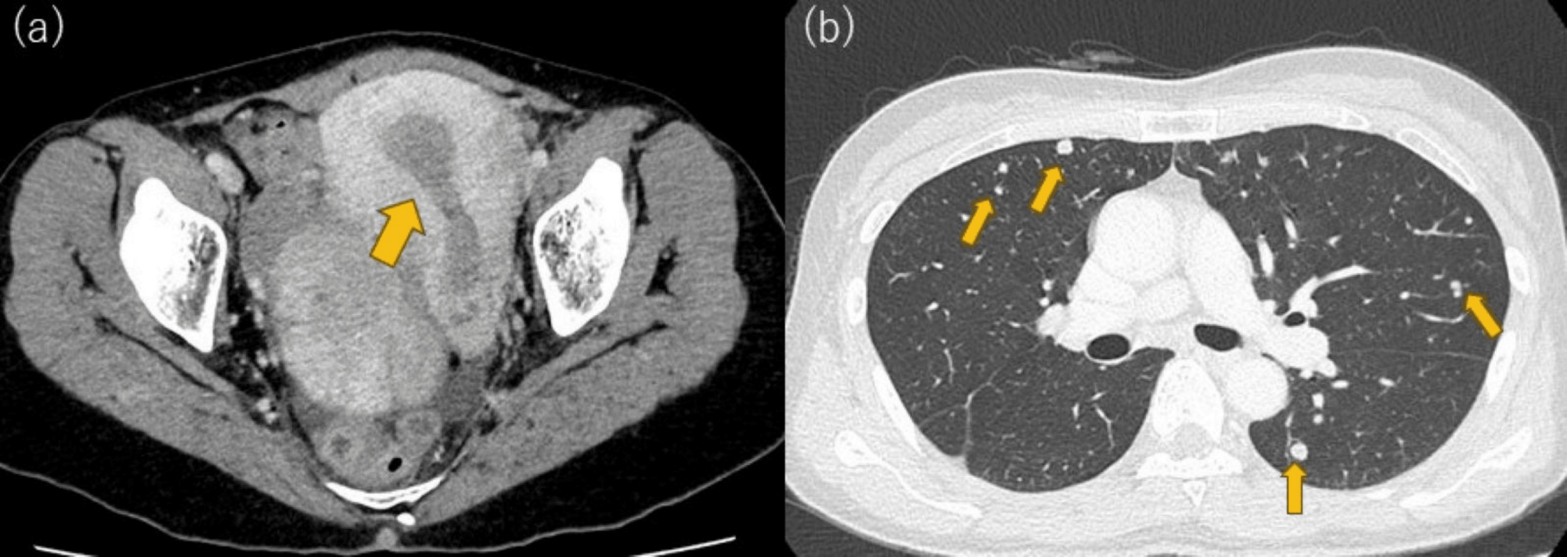
Contrast-enhanced CT showing endometrial thickening (a) and multiple pulmonary nodules consistent with metastases (b) (yellow arrows) CT: computed tomography

**Figure 2:**
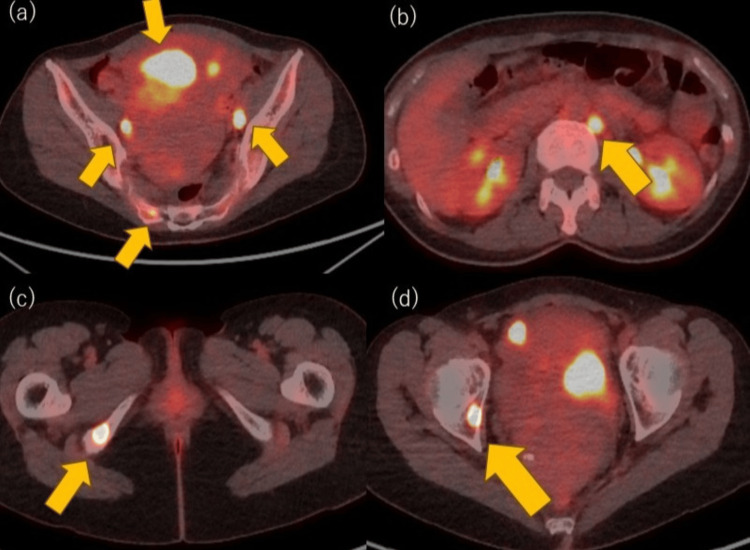
PET-CT images demonstrating multiple areas of increased uptake throughout the body: (a) uterus, pelvic lymph nodes, and sacrum; (b) para-aortic lymph nodes; (c) ischium; (d) acetabulum (yellow arrows) PET-CT: Positron emission tomography-computed tomography

A 55-year-old nulliparous, postmenopausal woman with no significant medical or family history presented with abnormal uterine bleeding, abdominal pain, and back pain. Computed tomography (CT) revealed multiple pulmonary nodules, pelvic and para-aortic lymphadenopathy, and endometrial thickening (Figure 1). Endometrial biopsy confirmed carcinoma. Pelvic magnetic resonance imaging (MRI) demonstrated a low endometrial signal on T2-weighted imaging, with restricted diffusion and myometrial invasion. Positron emission tomography-CT (PET-CT) showed abnormal uptake in the uterus, pelvic and para-aortic nodes, bilateral lungs, and right pelvic bones, consistent with metastatic endometrial cancer (Figure 2).

Neoadjuvant TC therapy was administered. After three cycles, the sizes of both the uterine and distant metastatic lesions decreased, and abnormal PET-CT uptake completely disappeared at all sites. The patient subsequently underwent total abdominal hysterectomy, bilateral salpingo-oophorectomy, omentectomy, and peritonectomy. Pathology confirmed endometrioid carcinoma grade 1, with no malignancy in the adnexa, omentum, or peritoneal deposits. Due to chemotherapy-induced peripheral neuropathy, adjuvant chemotherapy was switched to docetaxel-carboplatin (DC) for three cycles.

Two months after the final DC therapy, the patient developed pain while walking. Pelvic MRI revealed recurrent acetabulum metastases. Palliative radiotherapy (24 Gy/6 fractions) and lenvatinib plus pembrolizumab were initiated; however, the disease progressed. The patient was administered TC plus durvalumab.

**Figure 3:**
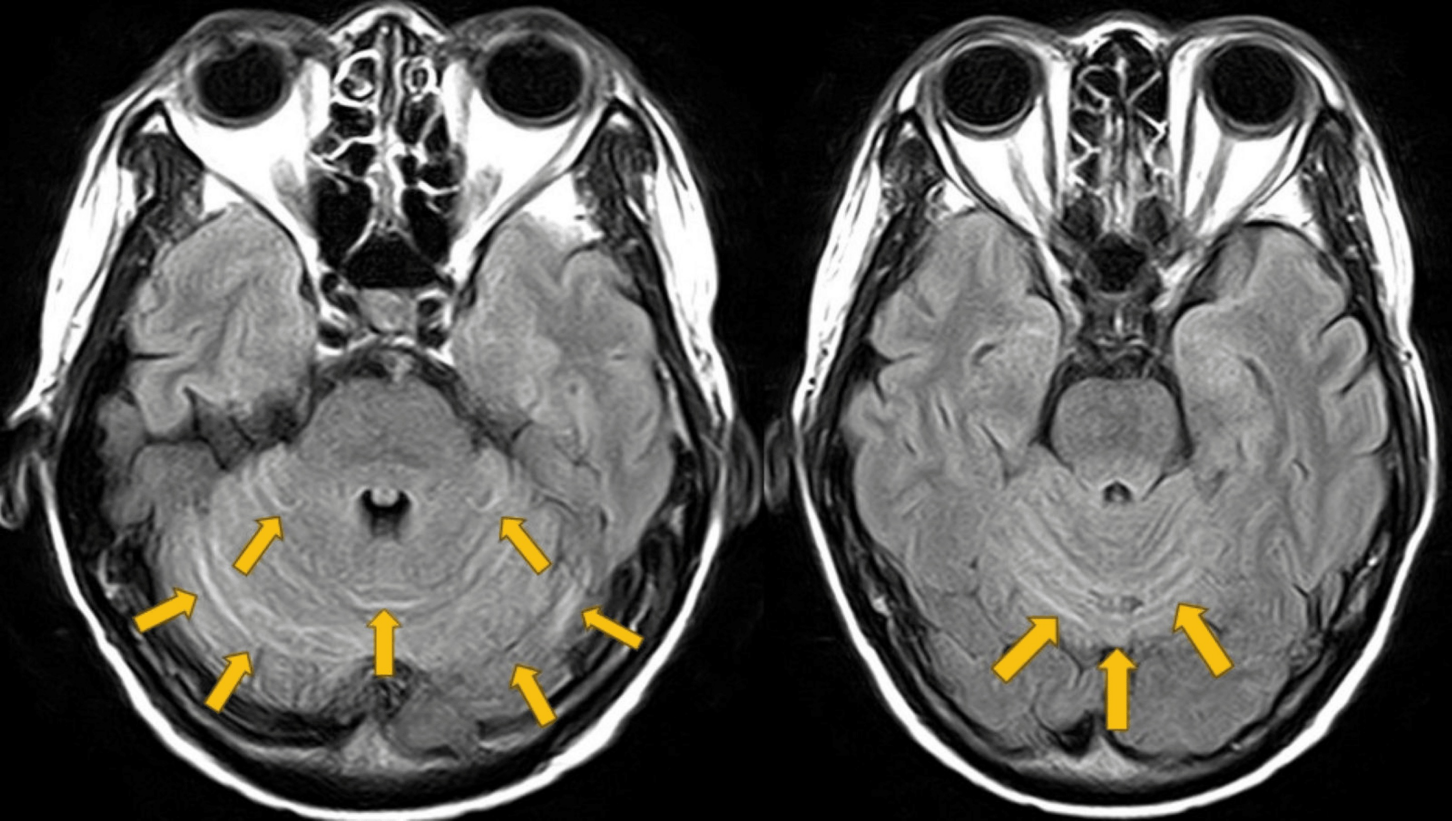
Contrast-enhanced T2-weighted MRI showing cerebellar high-signal intensity with prominent sulcal enhancement (yellow arrows) The observed contrast enhancement suggests the presence of inflammation in the affected area. MRI: magnetic resonance imaging

Six weeks after treatment initiation, the patient developed a sudden unsteady gait and was hospitalized. Brain MRI excluded stroke; however, her symptoms persisted over the next several days. The patient was transferred to a tertiary hospital for neurological support on the fifth day after symptom onset. Neurological examination revealed truncal ataxia, dizziness, dysarthria, and nystagmus. No significant muscle weakness or peripheral neuropathy was observed. Contrast-enhanced brain MRI revealed cerebellar enhancement, consistent with cerebellitis (Figure 3). Laboratory findings were unremarkable. Autoimmune antibody testing ruled out PNS (Table 1). Cerebrospinal fluid (CSF) analysis revealed pleocytosis, elevated protein levels, and positive oligoclonal bands, indicating CNS inflammation. The elevated lactate and adenosine deaminase levels also served as supportive indicators of underlying inflammation. CSF cytology and viral infection studies were negative (Table 2). Based on the clinical course, imaging findings, and CSF findings, immune-related cerebellitis secondary to durvalumab treatment was diagnosed.

**Table 1:** Autoantibody-related blood findings anti-Yo antibody: Purkinje cell cytoplasmic antibody type 1 (PCA-1), anti-Hu antibody: antineuronal nuclear antibody type 1 (ANNA-1), anti-Ri antibody: antineuronal nuclear antibody type 2 (ANNA-2), anti-CV2 antibody: collapsin response mediator protein 5 (CRMP5) antibody, anti-PNMA2 antibody: paraneoplastic Ma2/Ta (PNMA2) antibody, anti-SOX1 antibody: sex-determining region Y-box 1 (SOX1) antibody, anti-Zic4 antibody: zinc finger protein Zic4 antibody, anti-GAD65 antibody: glutamic acid decarboxylase 65 antibody, anti-Tr antibody: delta/notch-like epidermal growth factor-related receptor (DNER) antibody, anti-TPO: thyroid peroxidase antibody, PR3-ANCA: proteinase 3 antineutrophil cytoplasmic antibody, PNS: paraneoplastic neurological syndrome, MPO-ANCA: myeloperoxidase antineutrophil cytoplasmic antibody

Type	Results
Positive results	None
Negative results	PNS-related autoantibodies: anti-Yo, anti-Hu, anti-Ri, Anti-amphiphysin, anti-CV2, anti-PNMA, anti-SOX1, anti-recoverin, anti-titin, anti-zic4, anti-GAD65, and anti-Tr. Autoimmune disease-related antibodies: anti-nuclear, anti-SS-A/B, anti-thyroglobulin, anti-TPO, PR3-ANCA, and MPO-ANCA.

**Table 2:** CSF and serological findings An increase in CSF cell count and protein levels, along with the detection of oligoclonal bands, was observed. This suggested the onset of inflammation within the nervous system. ADA: adenosine deaminase, CSF: cerebrospinal fluid, HSV-IgG: herpes simplex virus-immunoglobulin G

Tests	Results	Reference range (typical)
Cell count (/μL)	16	<5
Protein (mg/dL)	69	15-45
Glucose (mg/dL)	57	50-75
Oligoclonal bands	Positive	Negative
Lactate (mmol/L)	19.6	1.2-2.1
Bilirubin (mg/dL)	1.2	<0.2
Adenosine deaminase, ADA (U/L)	12.4	<10
HSV-IgG	Negative	Negative
HSV-IgM	Negative	Negative

Steroid pulse therapy (1 g methylprednisolone for three days) was administered twice, resulting in partial improvement; however, nystagmus persisted, indicating residual neurological impairment. Intravenous immunoglobulin (IVIG) was administered with minimal benefit. Subsequently, the patient was switched to oral prednisolone (15 mg daily); nevertheless, symptoms persisted. Follow-up pelvic MRI showed regression of the metastatic lesions; however, owing to substantial deterioration in quality of life (QOL) related to ongoing neurological dysfunction, systemic therapy was discontinued, and the patient was transitioned to the best supportive care.

## Discussion

As a human monoclonal antibody against PD-L1, durvalumab prevents ligand interaction with PD-1 expressed on T lymphocytes, thereby restoring antitumor immune surveillance. In gynecological oncology, pembrolizumab has been approved for advanced or recurrent endometrial and cervical cancers, whereas cemiplimab has been introduced for recurrent cervical cancer progression after chemotherapy. Unlike cytotoxic chemotherapy or molecular-targeted agents, ICIs are associated with unique irAEs. Although approximately 90% of ICI-treated patients experience irAEs, neurological manifestations occur in just 5%, and severe grade ≥3 events in <1% [4]. Neurological irAEs typically appear around 6 weeks after treatment initiation [5]. Although the peripheral nervous system is more frequently affected, disruption of the blood-brain barrier allows activated T cells and ICIs to infiltrate the CNS, resulting in site-specific neurological symptoms [6]. Cerebellitis accounts for approximately 1% of neurological irAEs [7], with symptoms including dysarthria, nystagmus, oculomotor disturbances, ataxic gait, truncal ataxia, limb ataxia, dysmetria, and vertigo [3]. Affected individuals typically present with an acute or subacute onset of rapidly progressing cerebellar symptoms [6]. The analysis of CSF is crucial, as findings of elevated protein (>50 mg/dL), pleocytosis (>5 cells/mm³), and oligoclonal bands indicate CNS inflammation [3]. Brain MRI findings vary; however, T2/fluid-attenuated inversion recovery hyperintensities, edema, and gadolinium enhancement have been reported [8]. A systematic review of ICI-related cerebellitis showed a higher prevalence in men (63% vs. 37%), most frequently in non-small cell lung cancer (28.2%), followed by melanoma and Merkel cell carcinoma [8]. PD-1/PD-L1 inhibitors are more frequently associated with cerebellitis than CTLA-4 inhibitors [8]. Although durvalumab-related cerebellitis has been described in non-small cell lung cancer [9], it remains unreported in endometrial cancer, likely reflecting the more recent introduction of ICIs in gynecologic oncology.

The primary differential diagnosis was PNS. Paraneoplastic cerebellar ataxia presents with immune-mediated cerebellar dysfunction and is commonly associated with breast, ovarian, and uterine cancers [10]. The detection of paraneoplastic antibodies, particularly anti-Yo antibodies, is supportive but not mandatory, as their absence does not exclude a diagnosis [11]. The management of neurological irAEs relies on immunosuppression with corticosteroids, IVIG, or plasmapheresis. While grade 1 neurological irAEs may allow continuation of ICIs under close monitoring, grade ≥2 generally requires caution, and grade ≥3 necessitates permanent discontinuation [12]. The risk factors for refractory or relapsing neurological irAEs include older age, advanced cancer, severe initial presentation, myocarditis, CNS involvement, paraneoplastic antibodies, and autoimmune comorbidities [6]. For resistant cases, second-line therapies, such as rituximab, cyclophosphamide, mycophenolate mofetil, azathioprine, tocilizumab, or abatacept, may be considered [6].

In the present case, the diagnosis of immune-related cerebellitis was supported by clinical symptoms, MRI findings, and inflammatory CSF changes, along with negative paraneoplastic antibodies and the exclusion of infections and metastasis. The onset six weeks after initiation of durvalumab therapy further supported this causal association. Despite tumor regression in bone metastases, severe neurological toxicity led to treatment discontinuation and transition to supportive care, underscoring the devastating impact of neurological irAEs on an otherwise effective therapy. Given the suboptimal response to first-line therapies with high-dose corticosteroids and IVIG, second-line immunosuppressive agents such as rituximab might have been considered, provided informed consent was obtained. In this case, the presence of CNS involvement and advanced cancer might have predisposed the patient to resistance to immunosuppressive therapy, which could explain the insufficient response to first-line treatment.

This case emphasizes the need for heightened vigilance when administering durvalumab in gynecologic oncology. Routine neurological assessment, early neuroimaging, and timely CSF evaluation are essential for patients with even subtle neurological complaints. Early multidisciplinary collaboration with neuro-oncology specialists enables prompt diagnosis and immunosuppressive therapy, helping to prevent irreversible sequelae and preserve both QOL and treatment options.

## Conclusions

The incidence of neurological irAEs may increase with broader ICI use in gynecologic oncology. Therefore, a greater awareness of irAE-related neurological inflammation is essential. Specifically, when neurological symptoms appear shortly after ICI initiation, irAEs should be promptly considered and thoroughly investigated. As delays in treating neurological inflammation can cause irreversible sequelae and significant deterioration in QOL, early collaboration with neurologists is essential, as demonstrated by the partial recovery observed after timely immunosuppressive treatment in this case.

## References

[1] Westin SN, Moore K, Chon HS (2024). Durvalumab plus carboplatin/paclitaxel followed by maintenance durvalumab with or without olaparib as first-Line treatment for advanced endometrial cancer: the phase Ⅲ DUO-E trial. J Clin Oncol.

[2] Wei SC, Duffy CR, Allison JP (2018). Fundamental mechanism of immune checkpoint blockade therapy. Cancer Discov.

[3] Dentoni M, Florean I, Farina A (2024). Immune checkpoint inhibitor-related cerebellar toxicity: clinical features and comparison with paraneoplastic cerebellar ataxia. Cerebellum.

[4] Cuzzubbo S, Javeri F, Tissier M (2017). Neurological adverse events associated with immune checkpoint inhibitors: review of the literature. Eur J Cancer.

[5] Martins F, Sofiya L, Sykiotis GP (2019). Adverse effects of immune-checkpoint inhibitors: epidemiology, management and surveillance. Nat Rev Clin Oncol.

[6] Stavropoulou De Lorenzo S, Andravizou A, Alexopoulos H (2024). Neurological immune-related adverse events induced by immune checkpoint inhibitors. Biomedicines.

[7] Marini A, Bernardini A, Gigli GL, Valente M, Muñiz-Castrillo S, Honnorat J, Vogrig A (2021). Neurological adverse events if immune checkpoint inhibitors: a systematic review. Neurology.

[8] Dinoto A, Mantovani E, Ferrari S, Mariotto S, Tamburin S (2023). Cerebellar involvement associated with immune checkpoint inhibitors: a systematic review. Eur J Neurol.

[9] Connie T, Goran R (2020). Severe cerebellar syndrome linked with durvalumab. J Neurol Res.

[10] Afzal S, Recio M, Shamim S (2015). Paraneoplastic cerebellar ataxia and the paraneoplastic syndromes. Proc (Bayl Univ Med Cent).

[11] Hasadsri L, Lee J, Wang BH, Yekkirala L, Wang M (2013). Anti-yo associated paraneoplastic cerebellar degeneration in a man with large cell cancer of the lung. Case Rep Neurol Med.

[12] Schneider BJ, Naidoo J, Santomasso BD (2021). Management of immune-related adverse events in patients treated with immune checkpoint inhibitor therapy. J Clin Oncol.

